# Complete Genome Sequences of Two Marine Biofilm Isolates, *Leisingera* sp. nov. Strains 201A and 204H, Novel Representatives of the *Roseobacter* Group

**DOI:** 10.1128/MRA.00505-20

**Published:** 2020-07-09

**Authors:** Giselle S. Cavalcanti, Jessica Wasserscheid, Ken Dewar, Nicholas J. Shikuma

**Affiliations:** a Department of Biology, San Diego State University, San Diego, California, USA; b Viral Information Institute, San Diego State University, San Diego, California, USA; c Energy, Mining and Environment, National Research Council Canada, Montreal, Quebec, Canada; d Department of Human Genetics, McGill University, Montreal, Quebec, Canada; e McGill University and Genome Quebec Innovation Centre, McGill University, Montreal, Quebec, Canada; University of Maryland School of Medicine

## Abstract

Here, we report the complete-genome assemblies of biofilm isolates 201A and 204H. They possess six and seven plasmids, respectively, with a size ranging from 44 kb to 159 kb. Genomic comparisons place the two strains into one new species belonging to the genus *Leisingera* as novel representatives of the *Roseobacter* group.

## ANNOUNCEMENT

**FIG 1:**
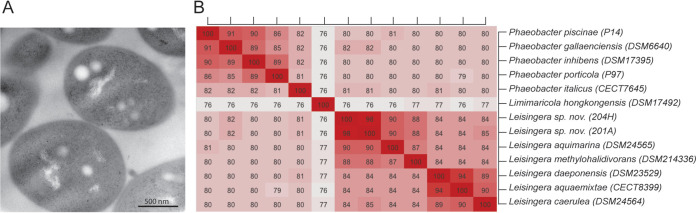
(A) Transmission electron micrograph of *Leisingera* sp. nov. strain 201A. (B) Average nucleotide identity (ANI) genome-based distance matrix of *Leisingera* sp. strains 201A and 204H relative to available *Leisingera* species genomes and closely related *Roseobacter* species. All values are given in percentages.

The novel isolates 201A and 204H were originally isolated from biofilms near tubes of the polychaete Hydroides elegans (Marina del Rey, CA, USA). Samples were cultured in artificial seawater tryptone (ASWT) agar medium (1) and incubated at 28°C for 48 h. The genome sequences of the isolates 201A and 204H place the two strains into one novel species within the genus *Leisingera* (Fig. 1), belonging to the *Roseobacter* lineage (*Rhodobacteraceae*), a widespread *Alphaproteobacteria* group (2, 3). Other *Roseobacter* isolates were shown to induce the metamorphosis of *H. elegans* (4) and the coral Porites astroides (5). These novel isolates recovered from biofilms near tubeworms, along with their relatedness to bacterial species known to induce animal metamorphosis, present interesting future investigation targets (6).

Genomic DNA from isolates 201A and 204H was extracted with a Zymo fungal/bacterial DNA miniprep kit and submitted to McGill University and Genome Quebec Innovation Centre (Montreal, Quebec, Canada) for Pacific Biosciences RS II sequencing (7). The DNA libraries were prepared following the Pacific Biosciences 20-kb template preparation with the SMRTbell template prep kit 1.0 reagents (Pacific Biosciences, Menlo Park, CA, USA). Large-insert DNA (≥20 kb) was sheared using g-TUBEs (Covaris, Inc., Woburn, MA, USA) and size selected using a BluePippin system (Sage Science, Inc., Beverly, MA, USA) following the manufacturer’s recommendations. Libraries were sequenced using the DNA sequencing kit 4.0 v2 and single-molecule real-time (SMRT) cells v3. Reads were filtered and assembled using SMRT Analysis v2.3 (PacBio) with default settings. We obtained a total of 77,942 reads covering a total of 797 Mb for isolate 201A and 70,939 reads totaling 709 Mb for isolate 204H. The mean subread length and *N*_50_ value were 8,299 bp and 12,584 bp, respectively. The final assembly performed using the HGAP workflow (8) resulted in a gapless circular chromosome (4,382,007 nucleotides [nt]) and six different closed circular plasmids (159,657 nt, 144,623 nt, 137,742 nt, 128,224 nt, 113,581 nt, and 71,922 nt) for strain 201A and a circular chromosome (4,246,063 nt) and seven associated gapless circular plasmids (151,185 nt, 148,812 nt, 140,681 nt, 124,119 nt, 122,006 nt, 59,982 nt, and 44,085 nt) for strain 204H. For each contig, ends were compared using BLAST (9) to (i) identify overlaps and (ii) confirm the uniqueness of the sequence to that locus. Edges were trimmed manually. The 5.1-Mb genomes had a total GC content of 62% with 72 RNAs for both strains. Annotation was performed with the Rapid Annotations using Subsystems Technology (RAST) server (10). RAST predicted 5,606 and 5,188 coding sequences for isolates 201A and 204H, respectively.

The novel isolates 201A and 204H had less than a 95% average amino acid identity/average nucleotide identity (AAI/ANI) and less than a 70% genome-to-genome distance (GGDH) from their closest neighbor, Leisingera methylohalidivorans (11, 12) (Fig. 1). The cutoffs used for the delimitation of bacterial species are more than 95% AAI and more than 70% GGDH (13). We conclude that the two isolates belong to a novel species of the genus *Leisingera.*

### Data availability.

The genome sequencing and assembly projects for *Leisingera* sp. nov. strains 201A and 204H have been deposited in DDBJ/EMBL/GenBank under accession number PRJNA515513. The raw sequencing data have been deposited under SRA accession numbers SRS6579670 and SRS6579671, respectively.
